# PD-L1 expression patterns in stage IB1 cervical squamous cell carcinoma: a retrospective study on implications for tumor budding and immune microenvironment

**DOI:** 10.7717/peerj.21052

**Published:** 2026-04-22

**Authors:** Shanming Lu, Kun Liu, Wenjuan Luo, Shaoqiu Zheng

**Affiliations:** 1 Department of Pathology, Longgang Central Hospital, Shenzhen, Guangdong, China; 2 Department of Pathology, Meizhou People’s Hospital, Meizhou, Guangdong, China

**Keywords:** Cervix, Squamous cell carcinoma, Tumor budding, Lymph node metastasis, PD-L1, CD8, FOXP3

## Abstract

**Background:**

The immune microenvironment at the tumor invasion front plays a pivotal role in cancer progression. Tumor budding, an aggressive histopathological feature, has been linked to poor outcomes in various malignancies. However, its impact on the tumor immune microenvironment remains poorly understood. This retrospective study aimed to determine whether tumor budding is a risk factor for lymph node metastasis (LNM) in stage IB1 cervical squamous cell carcinoma (CSCC) and to explore the relationship between PD-L1 expression in tumor cells and CD8^+^ and FOXP3^+^ tumor-infiltrating lymphocytes (TILs) within tumor budding regions.

**Methods:**

Tumor budding was retrospectively evaluated in 106 cases of International Federation of Gynecology and Obstetrics (FIGO) (2009) stage IB1 CSCC. High-grade tumor budding was defined as ≥ 15 buds per 10 high-power fields (HPFs). Immunohistochemistry was used to detect PD-L1, CD8, and FOXP3 expression. CD8^+^ T cells and FOXP3^+^ regulatory T cells (Tregs) were quantified separately intraepithelial and stromal compartments.

**Results:**

High-grade tumor budding was identified in 48 cases (45.3%) and was significantly associated with lymph node metastasis (LNM) on univariate analysis; however, it was not an independent predictor of LNM on multivariable logistic regression analysis. PD-L1 expression was detected in 53.8% (57/106) of the cohort, with overexpression observed in tumor budding areas. Three distinct PD-L1 expression patterns were identified: diffuse, marginal/tumor budding (MT), and negative. Notably, tumors with MT PD-L1 expression exhibited more favorable clinicopathological features compared to those with diffuse PD-L1 expression, including smaller tumor size, well differentiation, and low-grade tumor budding. In terms of the tumor immune microenvironment, PD-L1-negative tumors exhibited sparse infiltration of CD8^+^ T cells and FOXP3^+^ Tregs, whereas tumors with the MT pattern showed abundant stromal infiltration of both cell types. In contrast, diffuse PD-L1 expression was associated with abundant stromal and intraepithelial infiltration of CD8^+^ T cells and FOXP3^+^ Tregs.

**Conclusions:**

High-grade tumor budding is associated with LNM in stage IB1 CSCC. Three distinct PD-L1 expression patterns within tumor budding areas are associated with clinicopathologic features and immune cell infiltration profiles. PD-L1 expression patterns may warrant further investigation as potential biomarkers for immunotherapy response.

## Introduction

Cervical cancer is a prevalent malignancy of the female reproductive system. With the widespread implementation of cervical cancer screening programs, an increasing number of cases are being diagnosed at an early stage. International Federation of Gynecology and Obstetrics (FIGO) (2009) stage IB1 cervical cancer exhibits marked prognostic heterogeneity (Huang & Fang, 2018; Olthof et al., 2022). Patients with lymph node metastasis (LNM) have a poor prognosis, underscoring the urgency of identifying useful predictive biomarkers and therapeutic targets for LNM (Nanthamongkolkul & Hanprasertpong, 2018; Xu et al., 2022).

Tumor budding, an aggressive histopathological feature within the tumor microenvironment, has emerged as a predictor of LNM in several early-stage cancer (Pai et al., 2017; Jesinghaus et al., 2017; Du et al., 2019; Lin, Zamuco & Shukla, 2021). High-grade tumor budding has been documented to correlate significantly with LNM and poor prognosis in cervical cancer (Jesinghaus et al., 2018; Cao et al., 2020; Zare et al., 2020). The PD-1/PD-L1 pathway is a critical mediator of the tumor immunosuppressive microenvironment (Lin et al., 2024; Wang et al., 2024). Studies have shown that both tumor cells and inflammatory cells in tumor budding areas exhibit PD-L1 overexpression (Martinez-Ciarpaglini et al., 2019; Lang-Schwarz et al., 2021; Giatromanolaki et al., 2023). However, previous studies have primarily focused on the overall expression of PD-L1 or the independent role of tumor budding in cervical cancer, with limited attention paid to PD-L1 expression patterns specifically within tumor budding regions or their functional correlations with immune cell infiltration in CSCC. This gap limits our understanding of the interplay between tumor budding, PD-L1 spatial distribution, and the immune microenvironment in early-stage CSCC. Exploring the tumor immune microenvironment of tumor budding zones could enhance our understanding of tumor invasion and metastasis mechanisms.

This retrospective study aims to clarify the association between tumor budding and LNM in stage IB1 cervical squamous cell carcinoma and to explore the relationship between PD-L1 expression patterns and tumor-infiltrating lymphocytes (TILs) in tumor budding areas.

## Materials and Methods

### Patient cohort

The clinicopathological data of FIGO (2009) stage IB1 CSCC were retrospectively collected from 106 patients who underwent radical hysterectomy with pelvic lymphadenectomy at Meizhou People’s Hospital between 2010 and 2021. Patients were excluded if they met either criterion: (1) insufficient tumor tissue to assess tumor budding in ≥10 high-power fields (HPF); (2) prominent inflammation that obscured accurate assessment of tumor budding. In this study, 61 consecutive patients with pathologically confirmed LNM who met these criteria were included. As a control group, 45 consecutive patients without LNM who underwent surgery at the same hospital in 2018 were also enrolled. All included patients underwent systematic pelvic lymphadenectomy, and lymph node status was confirmed by histopathological examination. A detailed flow diagram illustrating patient selection is provided in  Fig. S1 (Supplementary Materials). All archived slides were independently reviewed by S.L. and S.Z. Tumor staging was determined according to the FIGO (2009) classification. This study was authorized by the Ethics Committee of Meizhou People’s Hospital (approval no. 2022-C-95). Given the retrospective nature of the study utilizing archived pathological specimens and anonymized data, and the extended time span (2010–2021) which could render obtaining individual written informed consent impractical for deceased or lost-to-follow-up patients, the requirement for written informed consent was waived by the Ethics Committee. All procedures were performed according to the World Medical Association’s Declaration of Helsinki.

### Assessment of tumor budding

All archived slides were reviewed, and those containing abundant tumor buds at the tumor invasion front were selected for analysis. Tumor budding was evaluated as previously described (Jesinghaus et al., 2018; Zare et al., 2020). Briefly, hotspot areas containing tumor buds at the invasive front were observed at 100 × magnification. The maximum number of tumor buds was calculated in 10 high-power fields (HPF; 0.275 mm^2^ per HPF). Tumor budding was graded as low-grade (0–14 buds) or high-grade (≥15 buds). Counts were independently performed by S.L. and S.Z. without knowledge of the patient’s clinical status. The inter-observer reliability for classifying tumors into low-grade or high-grade budding was substantial (Cohen’s kappa = 0.828) (Table S1). Discrepancies were resolved *via* joint discussion until a consensus was reached.

### Tissue microarray construction

Tissue microarrays were constructed from paraffin blocks matching the H&E slides used for tumor budding assessment. Punch cores (two mm in diameter) were sampled from hotspot areas containing tumor buds at the invasive front. Serial sections were used for H&E staining and immunohistochemical analysis.

### Immunohistochemistry

Tissue sections were deparaffinized, and antigen retrieval was performed using a high-pH citrate buffer. Immunohistochemical staining was conducted using a Ventana BenchMarkXT stainer (Roche, Basel, Switzerland) with the following monoclonal antibodies: anti-PD-L1 (SP263, prediluted; VENTANA, USA), anti-CD8 (MX020, prediluted; MXB, Jiangmen City, China), and anti-FOXP3 (236A/E7, 1:500; Abcam, Cambridge, UK). Immunostaining assessment was independently performed by S.L. and K.L. PD-L1 positivity was defined as complete or partial membranous staining in ≥1% of tumor cells, irrespective of intensity. PD-L1 expression pattern classification was independently conducted by two pathologists (S.L. and K.L.), who were blinded to the patients’ clinical status to eliminate potential bias. The inter-observer reliability for categorizing PD-L1 into the three expression patterns (negative, MT, and diffuse) was evaluated using Cohen’s kappa coefficient, yielding a substantial level of agreement (*κ* = 0.883); detailed consistency data are summarized in Table S2. CD8^+^ T cells and FOXP3^+^ regulatory T cells (Tregs) were counted separately within the tumor epithelium and stroma.

### Statistics

Pearson’s *χ*^2^ tests was used to analyze the correlations between tumor budding, clinicopathological parameters, and protein markers. A multivariable logistic regression analysis was then performed to identify independent predictors of LNM. The Kruskal–Wallis test was used to assess the correlation between PD-L1 expression patterns and immune-cell density. Given the multiple comparisons performed, Benjamini correction was applied to control for type I error. All statistical analyzes were performed using SPSS 23.0 (IBM SPSS Inc., Armonk, NY, USA), with *P* < 0.05 (two-tailed) considered statistically significant.

## Results

### Clinicopathological characteristics of the study cohort

**Figure 1:**
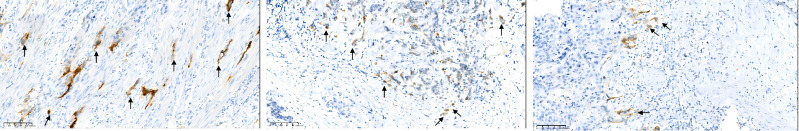
Immunohistochemical staining of PD-L1. PD-L1 overexpression was observed on tumor budding in different cases.

This study included 106 patients with FIGO stage IB1 CSCC, and their clinicopathologic features are summarized in Table 1. The median age of the patients was 48.52 years (range, 28–74 years). Tumor sizes ranged from 0.4 to 6.5 cm, with a median of 2.91 cm. Specifically, there were 26 tumors <2 cm, 60 tumors 2–4 cm, and 20 tumors ≥4 cm. Invasion depth was classified as superficial (<1/3 stromal invasion) in 26 cases, intermediate (1/3–2/3 stromal invasion) in 13 cases, and deep (>2/3 stromal invasion) in 67 cases. LNM was present in 61 cases, while none had parametrial invasion. The median number of tumor buds was 13 (range, 0–81), with 58 cases (54.7%) classified as low-grade tumor budding and 48 cases (45.3%) as high-grade tumor budding (Fig. 1). Most conventional unfavorable clinicopathological parameters were significantly associated with LNM in univariable analysis; however, only age, tumor size, and lymphovascular invasion (LVI) remained independent predictors of LNM in multivariable analysis. High-grade tumor budding was also significantly associated with LNM, but it did not constitute an independent predictor in the multivariable model.

**Table 1:** Association between clinicopathological features and LNM.

Clinicopahtological features	LNM, *n* (%)		*P-* value	Multivariable analysis		
	Absent	Present		*OR*	*95% CI*	**P*-* value
Age (y)			0.011	0.255	0.096–0.681	0.006
<49	16 (35.6)	37 (60.7)				
≥49	29 (64.4)	24 (39.3)				
Tumor size (cm)			0.001	9.538	1.569–57.983	0.014
<2 (T1b1)	18 (40.0)	8 (13.1)				
2∼4 (T1b2)	24 (53.3)	36 (59.0)				
≥4 (T1b3)	3 (6.7)	17 (27.9)				
Tumor grade			0.037	2.344	0.467–11759	0.300
G1/G2	42 (93.3)	48 (78.7)				
G3	3 (6.7)	13 (21.3)				
Invasion depth			0.002	1.473	0.491–4.419	0.489
<2/3 stromal invasion	24 (53.3)	15 (24.6)				
≥2/3 stromal invasion	21 (46.7)	46 (75.4)				
LVI			0.000	3.843	1.399–10.555	0.009
Absent	26 (57.8)	14 (23.0)				
Present	19 (42.2)	47 (77.0)				
Tumor budding			0.022	2.190	0.820–5.853	0.118
Low-grade	30 (66.7)	27 (44.3)				
High-grade	15 (33.3)	34 (55.7)				
PD-L1 patterns			0.034			
MT pattern	19	14				
Diffuse/Negative	26	47				

**Notes.**

Abbreviations LNM lymph node metastasis LVI lymphovascular invasion

### PD-L1 expression in tumor tissues

**Figure 2:**
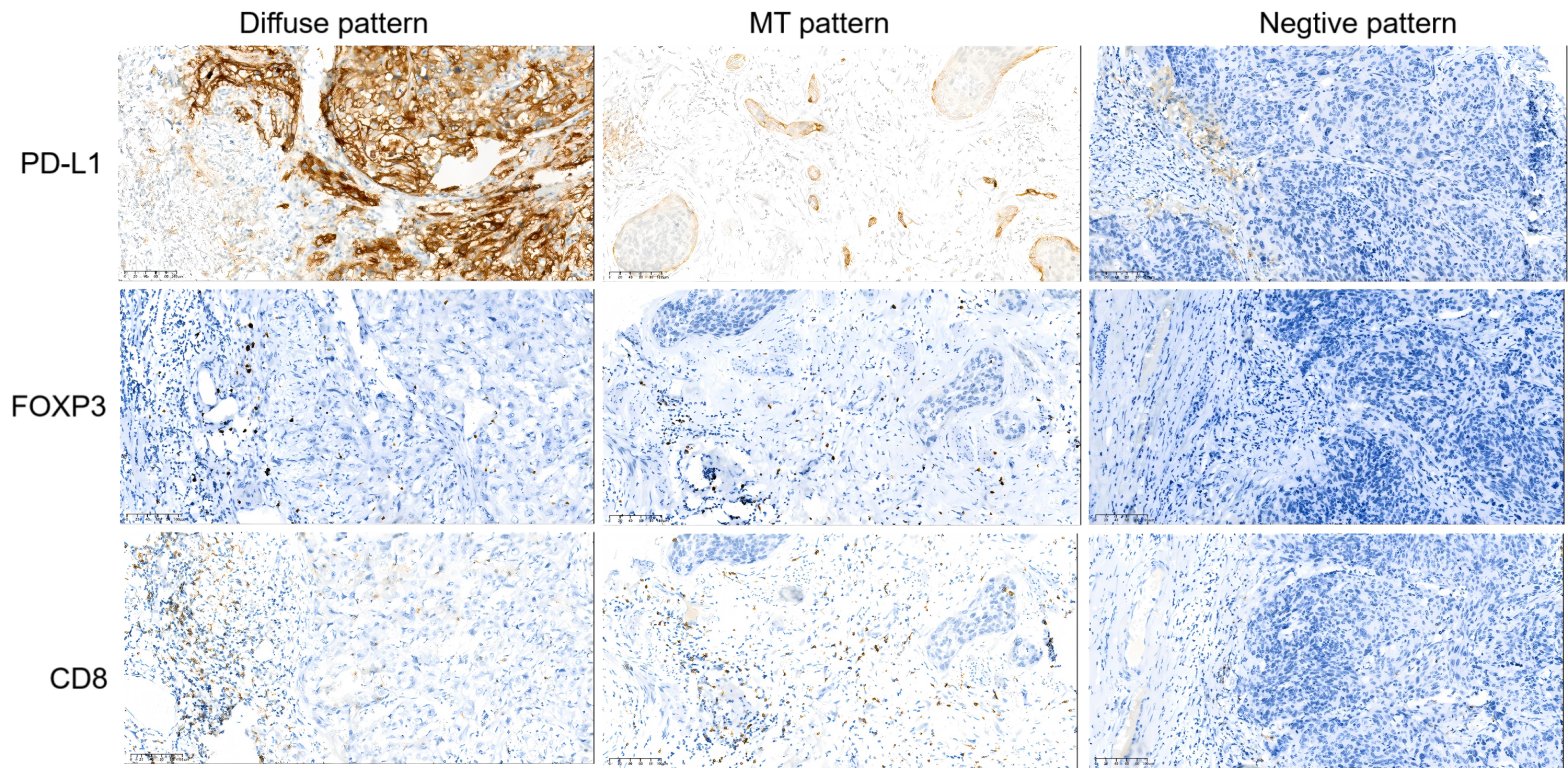
PD-L1 expression patterns and corresponding immune cell infiltration profiles in tumor microenvironment. Diffuse pattern: Tumor cells show diffuse PD-L1 expression, accompanied by abundant stromal and intraepithelial infiltration of CD8^+^T cells and FOXP3+ Treg cells. Marginal/tumor budding (MT) pattern: PD-L1 is restricted to the tumor-stroma interface and tumor buds, with marked stromal infiltration of CD8^+^T cells and FOXP3^+^ Tregs but minimal intraepithelial lymphocytic infiltration. Negative pattern: Tumor cells show no PD-L1 expression, with minimal infiltration of CD8^+^T cells and FOXP3^+^ Treg cells. Negative pattern: Tumor cells show no PD-L1 expression, associated with sparse infiltration of CD8^+^T cells and FOXP3^+^ Treg cells in both stroma and tumor nests.

PD-L1 expression was detected in tumor cells in 53.8% (57/106) of cases and was not significantly correlated with tumor budding or LNM (*P* > 0.05) (Table 2). Tumor buds were found to overexpress PD-L1 (Fig. 1). PD-L1 exhibited three distinct expression patterns in tumor cells: diffuse expression (22.7%), MT (marginal/tumor budding) expression (31.1%), and negative expression (46.2%) (Table 2). The MT expression pattern was defined as PD-L1 expression exclusively at the interface between tumor nests and stroma or on tumor buds, but not in the center of the tumor nests (Fig. 2). Compared with the diffuse pattern, cervical cancers exhibiting MT PD-L1 expression demonstrated more favorable clinicopathological features, including smaller tumor size, better histological differentiation, and lower-grade tumor budding. In contrast, tumors with MT PD-L1 expression showed clinicopathological characteristics similar to those of PD-L1–negative tumors.

**Table 2:** Association between clinicopathological features and immune cell markers with PD-L1 expression patterns.

Clinicopahtological features	PD-L1 expression n (%)		*P*-value	PD-L1 patterns n (%)		*P*-value	PD-L1 patterns n (%)		*P*-value	PD-L1 patterns n (%)		*P*-value
	Negative	Positive		Negative	Diffuse		MT	Diffuse		Negative	MT	
Age (y)			0.696			1.000			1.000			1.000
<49	23 (47.9)	30 (51.7)		23 (48.9)	15 (55.6)		15 (46.9)	15 (55.6)		23 (48.9)	15 (46.9)	
≥49	25 (52.1)	28 (48.3)		24 (51.1)	12 (44.4)		17 (53.1)	12 (44.4)		24 (51.1)	17 (53.1)	
Tumor size (cm)			0.700			0.858			0.021			0.288
<2	10 (20.8)	16 (27.6)		10 (21.3)	2 (7.4)		14 (43.7)	2 (7.4)		10 (21.3)	14 (43.7)	
2∼4	28 (58.4)	32 (55.2)		27 (57.4)	19 (70.4)		14 (43.8)	19 (70.4)		27 (57.4)	14 (43.8)	
≥4	10 (20.8)	10 (17.2)		10 (21.3)	6 (22.2)		4 (12.5)	6 (22.2)		10 (21.3)	4 (12.5)	
Tumor grade			0.339			1.000			0.033			0.168
G1/G2	39 (81.2)	51 (87.9)		39 (83.0)	20 (74.1)		31 (96.9)	20 (74.1)		39 (83.0)	31 (96.9)	
G3	9 (18.8)	7 (12.1)		8 (17.0)	7 (25.9)		1 (3.1)	7 (25.9)		8 (17.0)	1 (3.1)	
Invasion depth			0.282			1.000			0.339			0.318
<2/3 stromal invasion	15 (31.2)	24 (41.4)		15 (31.9)	8 (29.6)		16 (50.0)	8 (29.6)		15 (31.9)	16 (50.0)	
≥2/3 stromal invasion	33 (68.8)	34 (58.6)		32 (68.1)	19 (70.4)		16 (50.0)	19 (70.4)		32 (68.1)	16 (50.0)	
LNM			0.182			1.000			0.138			0.126
Absent	17 (35.4)	28 (48.3)		17 (36.2)	9 (33.3)		19 (59.4)	9 (33.3)		17 (36.2)	19 (59.4)	
Present	31 (64.6)	30 (51.7)		30 (63.8)	18 (66.7)		13 (40.6)	18 (66.7)		30 (63.8)	13 (40.6)	
LVI			0.118			0.441			1.000			0.501
Absent	22 (45.8)	18 (31.0)		22 (46.8)	8 (29.6)		10 (31.2)	8 (29.6)		22 (46.8)	10 (31.3)	
Present	26 (54.2)	40 (69.0)		25 (53.2)	19 (70.4)		22 (68.8)	19 (70.4)		25 (53.2)	22 (68.7)	
Tumor budding			0.642			0.138			0.039			1.000
Low-grade	27 (56.2)	30 (51.7)		27 (57.4)	9 (33.3)		21 (65.6)	9 (33.3)		27 (57.4)	21 (65.6)	
High-grade	21 (43.8)	28 (48.3)		20 (42.6)	18 (66.7)		11 (34.4)	18 (66.7)		20 (42.6)	11 (34.4)	

**Notes.**

Abbreviations LNM lymph node metastasis LVI lymphovascular invasion MT marginal/tumor budding iCD8/iFOXP3 intraepithelial CD8/FOXP3 cells sCD8/sFOXP3 stromal CD8/FOXP3 cells

Benjamini correction was applied to control for Type I error due to multiple comparisons.

### PD-L1 expression patterns and immune cell infiltration profiles

**Figure 3:**
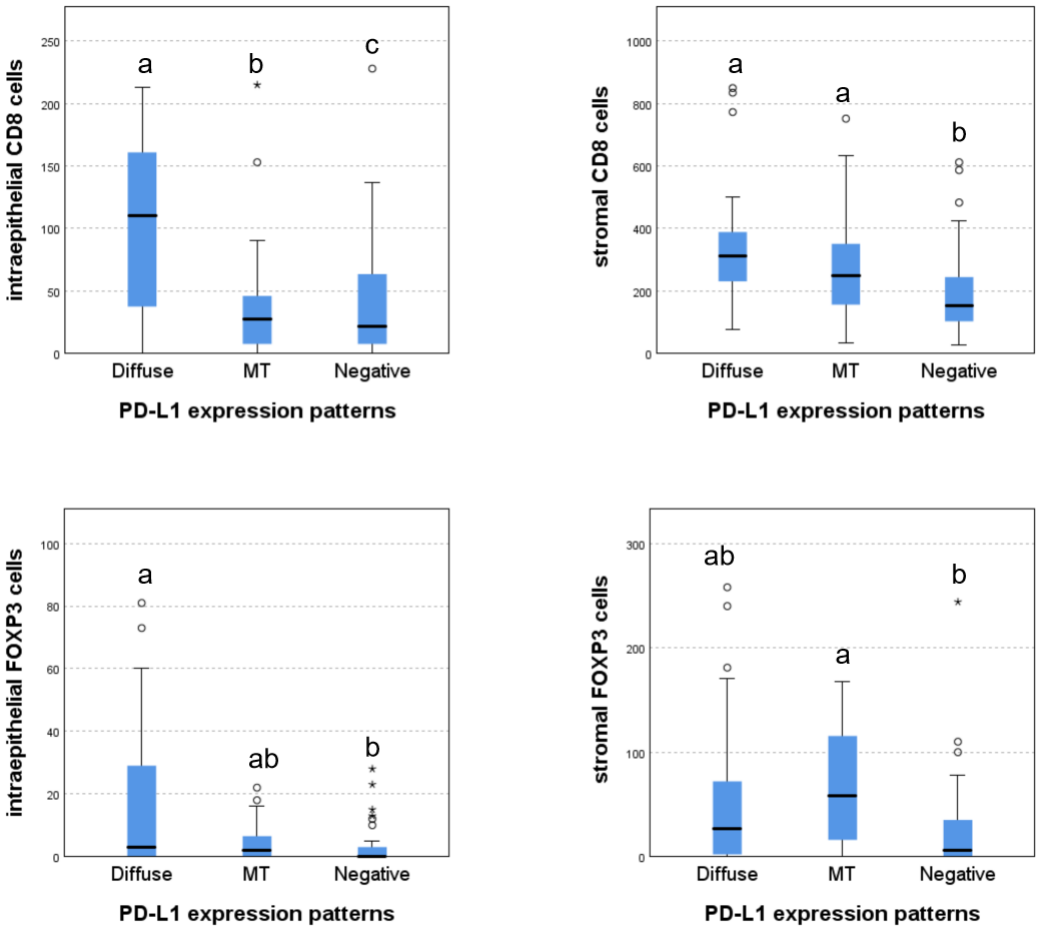
Immune-cell densities across distinct PD-L1 expression patterns. (A) Intraepithelial CD8 cells; (B) Stromal CD8 cells; (C) Intraepithelial FOXP3 cells; (D) Stromal FOXP3 cells; Differences between group labeled with different letters exhibited statistically significant.

Immune cells within the tumor epithelium and stroma were quantified separately. The median counts (with ranges) were as follows: intraepithelial CD8^+^ T cells, 56 (0–228); stromal CD8^+^ T cells, 259 (26–850); intraepithelial FOXP3^+^ Tregs, eight (0–81); and stromal FOXP3^+^ Tregs, 45 (0–258). PD-L1-expressing tumors exhibited greater immune cell infiltration compared with PD-L1-negative tumors. Specific immune cell infiltration profiles were associated with different PD-L1 expression patterns (Fig. 3). Tumors with negative PD-L1 expression hhad a low number of infiltrating CD8^+^ T cells and FOXP3^+^ Tregs. Tumors with MT PD-L1 expression demonstrated a richer stromal infiltration of CD8^+^ T cells and FOXP3^+^ Treg cells, while those with diffuse PD-L1 expression exhibited abundant stromal and intraepithelial infiltration of CD8^+^ T cells and FOXP3^+^ Treg cells (Fig. 2).

## Discussion

Tumor budding, an integral component of the tumor microenvironment, is a known predictor of LNM in cervical cancer (Jesinghaus et al., 2018; Cao et al., 2020; Zare et al., 2020). Using the same assessment method as described in previous reports (Jesinghaus et al., 2018; Zare et al., 2020), we confirmed that tumor budding is associated with LNM in stage IB1 CSCC. Therefore, using a threshold of ≥15 tumor buds per 10 high-power fields (400 × magnification) to define high-grade tumor budding can help assess the risk of LNM in cervical cancer. Consistent with previous reports (Nanthamongkolkul & Hanprasertpong, 2018; Xu et al., 2022), conventional adverse pathological factors such as large tumor size, poor differentiation, deep stromal invasion, and LVI were significantly associated with LNM in stage IB1 squamous cervical cancer supports the progression of tumor budding.

Tumor budding, a histological marker of epithelial-mesenchymal transition, confers migratory and invasive capabilities that drive tumor spread (Lugli et al., 2021). The tumor microenvironment (comprising cancer-associated fibroblasts, immune cells, and extracellular matrix) supports the progression of tumor budding, while tumor buds releases bioactive substances (*e.g.*, cytokines) to modulate the microenvironment, forming a reciprocal feedback loop (Gao et al., 2022; Lugli et al., 2021; Shakhpazyan et al., 2023; Hatthakarnkul et al., 2022). Notably, the PD-1/PD-L1 immunosuppressive axis is critical for tumor progression (Lin et al., 2024; Wang et al., 2024). PD-L1 overexpression has been observed in cervical precancerous lesions and cervical cancers (Mezache et al., 2015; Dominoni et al., 2024; Rotman et al., 2020). Our study demonstrated that PD-L1 was expressed in 53.8% of cervical cancers, a finding consistent with the report by Heeren et al. (2016). PD-L1 expression exhibits spatial heterogeneity, with higher expression in the tumor budding area at the invasive front compared with central tumor regions (Martinez-Ciarpaglini et al., 2019; Dominoni et al., 2024). Inconsistent findings across studies (Lang-Schwarz et al., 2021; Secinti, Ozgur & Dede, 2022; Baş et al., 2023) likely result from variations in PD-L1 assessment methods and tissue sampling sites. Our study extends previous research by systematically classifying PD-L1 expression patterns within tumor budding regions (diffuse, MT, negative) and linking these patterns to immune infiltration profiles, which has not been thoroughly explored in stage IB1 CSCC.

In this study, we sampled from tumor budding-rich areas to observe the spatial localization of PD-L1 relative to tumor budding and immune cells. The PD-L1 expression pattern showed a significant association with tumor budding and LNM. Tumors with diffuse PD-L1 expression exhibited the highest degree of tumor budding. Conversely, tumors with MT PD-L1 expression demonstrated more favorable clinicopathological features, including lower LNM rates and low-grade tumor budding compared to those with diffuse/negative PD-L1 expression. However, in a multivariate model that included established pathological risk factors, the PD-L1 expression pattern itself was not an independent predictor of LNM. These three PD-L1 expression patterns have also been observed in squamous cell carcinomas at other sites, such as the head and neck, penis, oral cavity, and anal canal (Heeren et al., 2016; Ottenhof et al., 2017; Succaria et al., 2021; Miranda-Galvis et al., 2021; Yanik et al., 2017). Marginal expression has been associated with better prognosis in these cancers and is more common in human papillomavirus (HPV)-associated tumors, which often exhibited pronounced inflammation (Heeren et al., 2016; Ottenhof et al., 2017; Succaria et al., 2021; Miranda-Galvis et al., 2021; Yanik et al., 2017). PD-L1 expression is controlled by intrinsic and extrinsic pathways (Yamaguchi et al., 2022). PD-L1 expression is also regulated by a pro-inflammatory tumor microenvironment (Lin et al., 2024; Jiang et al., 2019).

To characterize the immune microenvironment associated with PD-L1 expression, we analyzed CD8^+^T cells and FOXP3^+^ Tregs infiltration in stage IB1 CSCC with distinct PD-L1 expression patterns (Fig. 2). Key findings revealed that PD-L1 expression patterns in tumor budding areas were correlated with immune cell infiltration (Fig. 3): (1) Negative pattern: Minimal CD8^+^ T cell and FOXP3^+^ Treg infiltration; (2) MT pattern: Moderate stromal infiltration of both cell types; (3) Diffuse pattern: Abundant stromal and intraepithelial infiltration of both cell types. High PD-L1 expression and abundant immune cell infiltration serve as hallmarks of the tumor budding area and are linked to patient prognosis (Lang-Schwarz et al., 2021; Giatromanolaki et al., 2023; Dominoni et al., 2024; Georges et al., 2021). Lin et al. (2023) constructed a three-dimensional (3D) tumor microenvironment atlas for colorectal cancer, identifying that tumor budding regions exhibit the highest density of immune cells, particularly CD4^+^FOXP3^+^ Tregs, with some Tregs colocalizing with CD8^+^ cytotoxic T cells. Additionally, they found that in some colorectal cancers, PD-L1-expressing tumor cells are concentrated at the edges of tumor buds, adjacent to T cells. Guil-Luna et al. (2020) and colleagues observed that high-grade tumor budding zones are characterized by elevated expression of inhibitory immune-checkpoint molecules, Toll-like receptors, and an array of chemokine receptors and their ligands. These findings implicate a specialized immunosuppressive microenvironment that accompanies tumor budding. In our study, diffuse PD-L1 expression was associated with a highly immunosuppressive niche (rich in CD8^+^ T cells, FOXP3^+^ Tregs, and PD-L1^+^ buds), which may facilitate local tumor cell invasion and local dissemination. The MT pattern, with balanced immune infiltration and limited PD-L1^+^ buds, was correlated with favorable clinicopathologic features. While PD-L1- tumors, with reduced immune cell infiltration, displayed aggressive behavior similar to diffuse pattern tumors.

Collectively, PD-L1 expression patterns may reflect the immune microenvironment heterogeneity in tumor budding areas. This study had several limitations. First, the retrospective, single-institution design introduces potential selection bias, constrains causal inference, and may limit the generalizability (external validity) of our findings to other patient populations. Future prospective, multi-center studies are warranted to validate these findings. Second, the sample size (*n* = 106) was relatively small, potentially compromising statistical power and limiting generalizability. Third, the use of tissue microarrays (TMAs) with 2-mm punch cores, while efficient for high-throughput analysis, may lead to undersampling of the full spatial heterogeneity of PD-L1 expression and immune cell infiltration across entire tumor sections. However, given that our study specifically aimed to analyze the tumor budding regions at the invasive front, we sampled cores from these pre-identified hotspot areas. This targeted approach ensured that the key morphological and immunological features of interest were captured. Fourth, this study did not assess human papillomavirus (HPV) subtypes in the included cases, a limitation that warrants acknowledgment. HPV-18-associated cervical cancer is well documented to exhibit more aggressive biological behavior (Chen et al., 2020), which may be reflected in enhanced tumor budding activity, an increased risk of lymph node metastasis (LNM), and altered PD-1/PD-L1 expression profiles in the tumor microenvironment. The interaction between specific HPV subtypes, the spatial distribution of PD-L1 in tumor budding regions, and the subsequent modulation of immune cell infiltration remains to be elucidated. Growing evidence supports tumor budding as a hallmark of cancer progression (Lugli et al., 2021), with therapeutic strategies targeting this phenomenon gaining traction. PD-L1 expression in tumor buds may make PD-L1-targeted therapies promising for countering tumor progression.

## Conclusions

This study demonstrated that distinct PD-L1 expression patterns reflect immune microenvironment heterogeneity and show a significant association with LNM in stage IB1 cervical cancer. The findings highlight the clinical relevance of PD-L1 spatial distribution in reflecting tumor aggressiveness and immune cell infiltration profiles. Future research should prioritize prospective validation of these PD-L1 expression patterns and investigate their correlation with immunotherapy response in larger cohorts.

##  Supplemental Information

10.7717/peerj.21052/supp-1 Supplemental Information 1 Agreement of bud counts assessed by the two pathologists

10.7717/peerj.21052/supp-2 Supplemental Information 2 Agreement of PD-L1 expression patterns assessed by the two pathologists

10.7717/peerj.21052/supp-3 Supplemental Information 3 Patient flow diagram for cohort assembly Initial screening included 574 patients diagnosed with FIGO Stage IB1 cervical squamous cell carcinoma (CSCC) who were treated between 2010 and 2021. The lymph node metastasis (LNM)-positive group consisted of consecutive patients with pathologically confirmed LNM during 2010–2021 (n=66); 5 patients were excluded due to insufficient tumor tissue or severe inflammation, and 61 patients were finally included. The LNM-negative control pool was composed of patients without LNM from the 2018 sub-cohort (n=49); 4 patients were excluded based on the same exclusion criteria (insufficient tissue/severe inflammation), and 45 patients were enrolled in the LNM-negative control group.

10.7717/peerj.21052/supp-4 Supplemental Information 4 Raw data about clinicopathological features, immune cell markers and PD-L1 expression

10.7717/peerj.21052/supp-5 Supplemental Information 5 STROBE checklist

## Additional Information and Declarations

### Data Availability

The following information was supplied regarding data availability: The raw data are available in the Supplemental File.
