# Supplementary material for: PD-L1 expression patterns in stage IB1 cervical squamous cell carcinoma: a retrospective study on implications for tumor budding and immune microenvironment
Source: PeerJ. 2026 Apr 22;14:e21052. doi: 10.7717/peerj.21052 (PMC13109980; doi:10.7717/peerj.21052)
Supplement: Supplemental Information 1 [file peerj-14-21052-s001.docx]

| Pathologist 1 | Pathologist 2 | | Kappa | *P*-value |
| --- | --- | --- | --- | --- |
|  | Low-grade TB (n) | High-grade TB (n) |  |  |
| Low-grade TB | 55 | 2 | 0.828 | 0.000 |
| High-grade TB | 7 | 42 |  |  |
