# Supplementary material for: PD-L1 expression patterns in stage IB1 cervical squamous cell carcinoma: a retrospective study on implications for tumor budding and immune microenvironment
Source: PeerJ. 2026 Apr 22;14:e21052. doi: 10.7717/peerj.21052 (PMC13109980; doi:10.7717/peerj.21052)
Supplement: Supplemental Information 2 [file peerj-14-21052-s002.docx]

| Pathologist 1 | Pathologist 2 | | | Kappa | *P*-value |
| --- | --- | --- | --- | --- | --- |
|  | Negative | MT | Diffuse |  |  |
| Negative | 47 | 0 | 0 | 0.883 | 0.000 |
| MT | 0 | 28 | 4 |  |  |
| Diffuse | 0 | 4 | 23 |  |  |
