# Supplementary figures and images for: PD-L1 expression patterns in stage IB1 cervical squamous cell carcinoma: a retrospective study on implications for tumor budding and immune microenvironment
Source: PeerJ. 2026 Apr 22;14:e21052. doi: 10.7717/peerj.21052 (PMC13109980; doi:10.7717/peerj.21052)

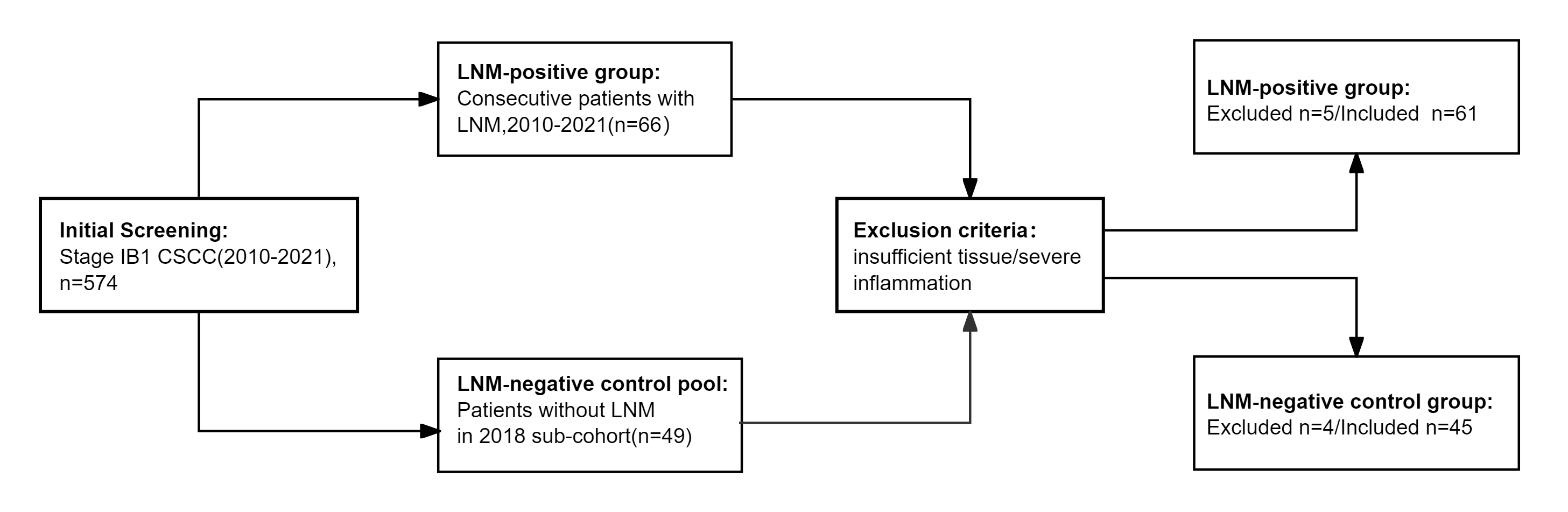

Supplement: Supplemental Information 3 — Initial screening included 574 patients diagnosed with FIGO Stage IB1 cervical squamous cell carcinoma (CSCC) who were treated between 2010 and 2021. The lymph node metastasis (LNM)-positive group consisted of consecutive patients with pathologically confirmed LNM during 2010–2021 (n=66); 5 patients were excluded due to insufficient tumor tissue or severe inflammation, and 61 patients were finally included. The LNM-negative control pool was composed of patients without LNM from the 2018 sub-cohort (n=49); 4 patients were excluded based on the same exclusion criteria (insufficient tissue/severe inflammation), and 45 patients were enrolled in the LNM-negative control group. [file peerj-14-21052-s003.png]
