# Supplementary material for: PD-L1 expression patterns in stage IB1 cervical squamous cell carcinoma: a retrospective study on implications for tumor budding and immune microenvironment
Source: PeerJ. 2026 Apr 22;14:e21052. doi: 10.7717/peerj.21052 (PMC13109980; doi:10.7717/peerj.21052)
Supplement: Supplemental Information 5 [file peerj-14-21052-s005.docx]

STROBE Statement—checklist of items that should be included in reports of observational studies

|  | Item No. | Recommendation | Page  No. | Relevant text from manuscript |
| --- | --- | --- | --- | --- |
| **Title and abstract** | 1 | (*a*) Indicate the study’s design with a commonly used term in the title or the abstract | 1 | The title includes "a retrospective study" and the abstract starts with "This retrospective study aims to...". |
|  |  | (*b*) Provide in the abstract an informative and balanced summary of what was done and what was found | 1 | The abstract summarizes that tumor budding was evaluated in 106 cases of FIGO (2009) stage IB1 CSCC, and immunohistochemistry was used to detect PD-L1, CD8 and FOXP3 expressions. Results showed high-grade tumor budding was associated with LNM, and PD-L1 had three expression patterns related to clinicopathologic features and immune cells infiltration. |
| Introduction | | | |  |
| Background/rationale | 2 | Explain the scientific background and rationale for the investigation being reported | 3 | Cervical cancer is a prevalent malignancy. FIGO (2009) stage IB1 cervical cancer has prognostic heterogeneity. Patients with LNM have poor prognosis. Tumor budding is a predictor of LNM in some cancers but its impact in stage IB1 CSCC is limited. The PD-1/PD-L1 pathway is critical in tumor immunosuppressive microenvironment, and PD-L1 is overexpressed in tumor budding areas. |
| Objectives | 3 | State specific objectives, including any prespecified hypotheses | 3 | To determine whether tumor budding is a risk factor for LNM in stage IB1 CSCC and to explore the relationship between PD-L1 expression in tumor cells and CD8⁺/FOXP3⁺ TILs within tumor budding areas. |
| Methods | | | |  |
| Study design | 4 | Present key elements of study design early in the paper | 3 | This is a retrospective study that evaluated tumor budding in 106 cases of FIGO (2009) stage IB1 CSCC, used immunohistochemistry to detect PD-L1, CD8 and FOXP3 expressions, and analyzed the correlations. |
| Setting | 5 | Describe the setting, locations, and relevant dates, including periods of recruitment, exposure, follow-up, and data collection | 3 | The study was conducted at Meizhou People's Hospital. Clinicopathological data were collected from patients who underwent surgery between 2010 and 2021. The control group was from patients who underwent surgery in 2018 at the same hospital. |
| Participants | 6 | (*a*) *Cohort study*—Give the eligibility criteria, and the sources and methods of selection of participants. Describe methods of follow-up  *Case-control study*—Give the eligibility criteria, and the sources and methods of case ascertainment and control selection. Give the rationale for the choice of cases and controls  *Cross-sectional study*—Give the eligibility criteria, and the sources and methods of selection of participants | 3 | Eligibility criteria were FIGO (2009) stage IB1 CSCC. Participants were selected from those who underwent surgery at Meizhou People's Hospital between 2010 and 2021. 61 consecutive patients with lymph node metastases and 45 patients without lymph node metastases from another consecutive cohort were included. All archived slides were reviewed by S.L. and S.Z. Patients without sufficient tumor tissue for assessment or with prominent inflammation were excluded. |
|  |  | (*b*) *Cohort study*—For matched studies, give matching criteria and number of exposed and unexposed  *Case-control study*—For matched studies, give matching criteria and the number of controls per case |  | Not a matched study. |
| Variables | 7 | Clearly define all outcomes, exposures, predictors, potential confounders, and effect modifiers. Give diagnostic criteria, if applicable | 4 | Outcomes: LNM,  Exposures: Tumor budding, PD-L1 expression patterns, CD8⁺ T cells and FOXP3⁺ Tregs infiltration.  Predictors: Tumor budding grade, PD-L1 expression patterns.  Diagnostic criteria: Tumor budding was graded as low-grade (0–14 buds) or high-grade (≥15 buds).PD-L1 positivity was defined as complete or partial membranous staining in ≥ 1% of tumor cells. CD8+ and FOXP3+ TILs positive expression criteria were specified (iCD8 ≥50; sCD8 ≥ 200; iFOXP3 ≥ 10; sFOXP3 ≥ 50). |
| Data sources/ measurement | 8* | For each variable of interest, give sources of data and details of methods of assessment (measurement). Describe comparability of assessment methods if there is more than one group | *4* | Tumor budding: Assessed from archived slides, counted in 10 HPF by S.L. and S.Z.  PD-L1, CD8, FOXP3: Detected by immunohistochemistry, assessed by S.L. and K.L.  Data sources were clinicopathological records and tissue samples.  No multiple groups with different assessment methods. |
| Bias | 9 | Describe any efforts to address potential sources of bias | 4 | Counts of tumor budding and immunostaining assessment were independently performed by two researchers without knowledge of the patient's clinical status, and discrepancies were resolved through discussion. |
| Study size | 10 | Explain how the study size was arrived at | 4 | The sample size of this retrospective study was determined based on the availability of eligible patient data that met predefined inclusion and exclusion criteria, rather than a prospectively calculated sample size using statistical formulas. |

Continued on next page

| Quantitative variables | 11 | Explain how quantitative variables were handled in the analyses. If applicable, describe which groupings were chosen and why | 4 | Tumor budding was graded as low-grade (0–14 buds) or high-grade (≥15 buds) based on the number of buds per 10 HPF. CD8+ and FOXP3+ TILs were classified into positive and negative based on specific cell counts. |
| --- | --- | --- | --- | --- |
| Statistical methods | 12 | (*a*) Describe all statistical methods, including those used to control for confounding | 5 | (a) Pearson χ² test was used to analyze correlations. |
|  |  | (*b*) Describe any methods used to examine subgroups and interactions | 5 | This study explored the differences among subgroups and their potential interactive associations through stratified comparisons and chi-square tests. |
|  |  | (*c*) Explain how missing data were addressed |  | No missing data |
|  |  | (*d*) *Cohort study*—If applicable, explain how loss to follow-up was addressed  *Case-control study*—If applicable, explain how matching of cases and controls was addressed  *Cross-sectional study*—If applicable, describe analytical methods taking account of sampling strategy |  | This study does not involve follow-up data. |
|  |  | (*e*) Describe any sensitivity analyses | 4 | This study ensured the robustness of the results through dual verification of key indicators and strict inclusion criteria. |
| Results | | | | |
| Participants | 13* | (a) Report numbers of individuals at each stage of study—eg numbers potentially eligible, examined for eligibility, confirmed eligible, included in the study, completing follow-up, and analysed | 5 | 106 cases of FIGO (2009) stage IB1 CSCC were included, with 61 in the lymph node metastasis group and 45 in the control group. All were analysed. |
|  |  | (b) Give reasons for non-participation at each stage | 3 | Patients without sufficient tumor tissue for assessment or with prominent inflammation were excluded. |
|  |  | (c) Consider use of a flow diagram |  |  |
| Descriptive data | 14* | (a) Give characteristics of study participants (eg demographic, clinical, social) and information on exposures and potential confounders | 5 | Median age was 48.52 years (range: 28–74 years). Tumor sizes ranged from 0.4 to 6.5 cm, median 2.91 cm. Invasion depth: superficial in 26, intermediate in 13, deep in 67. 61 had LNM, none had parametrial invasion. 58 had low-grade tumor budding, 48 high-grade. |
|  |  | (b) Indicate number of participants with missing data for each variable of interest | 5 | All cases were analysed. |
|  |  | (c) *Cohort study*—Summarise follow-up time (eg, average and total amount) |  | No follow-up data. |
| Outcome data | 15* | *Cohort study*—Report numbers of outcome events or summary measures over time | *5, 6* | High-grade tumor budding in 48 (45.3%) cases, significantly associated with LNM. PD-L1 expression in 57 (53.8%) cases, with three patterns. iCD8+ 27.4%, sCD8+ 62.3%, iFOXP+ 24.5%, sFOXP+ 32.1%.\| |
|  |  | *Case-control study—*Report numbers in each exposure category, or summary measures of exposure |  |  |
|  |  | *Cross-sectional study—*Report numbers of outcome events or summary measures |  |  |
| Main results | 16 | (*a*) Give unadjusted estimates and, if applicable, confounder-adjusted estimates and their precision (eg, 95% confidence interval). Make clear which confounders were adjusted for and why they were included | 5 | The study primarily utilized descriptive analyses and Pearson’s χ² test to explore associations between variables. |
|  |  | (*b*) Report category boundaries when continuous variables were categorized | 5 | In this study, several continuous variables were categorized into distinct groups based on specific boundaries, as detailed below:  Tumor size:  Categorized into three groups based on diameter:  < 2 cm  2–4 cm  ≥ 4 cm  Invasion depth:  Classified according to the proportion of stromal invasion:  Superficial: < 1/3 stromal invasion  Intermediate: 1/3–2/3 stromal invasion  Deep: > 2/3 stromal invasion  Tumor budding:  Graded based on the number of buds per 10 high-power fields (HPF):  Low-grade: 0–14 buds/10 HPF  High-grade: ≥ 15 buds/10 HPF  CD8⁺ and FOXP3⁺ tumor-infiltrating lymphocytes (TILs):  Categorized into positive and negative groups using threshold counts:  Intra-tumoral CD8⁺ T cells (iCD8): ≥ 50 cells (positive) vs. < 50 cells (negative)  Stromal CD8⁺ T cells (sCD8): ≥ 200 cells (positive) vs. < 200 cells (negative)  Intra-tumoral FOXP3⁺ Tregs (iFOXP3): ≥ 10 cells (positive) vs. < 10 cells (negative)  Stromal FOXP3⁺ Tregs (sFOXP3): ≥ 50 cells (positive) vs. < 50 cells (negative) |
|  |  | (*c*) If relevant, consider translating estimates of relative risk into absolute risk for a meaningful time period |  | Not relevant. |

Continued on next page

| Other analyses | 17 | Report other analyses done—eg analyses of subgroups and interactions, and sensitivity analyses |  | No other analyses. |
| --- | --- | --- | --- | --- |
| Discussion | | | | |
| Key results | 18 | Summarise key results with reference to study objectives | 6 | High-grade tumor budding is a risk factor for LNM in stage IB1 CSCC. Tumor buds overexpress PD-L1. Three PD-L1 expression patterns are associated with clinicopathologic features and immune cell infiltration, which may be potential biomarkers for immunotherapy response. |
| Limitations | 19 | Discuss limitations of the study, taking into account sources of potential bias or imprecision. Discuss both direction and magnitude of any potential bias | 8 | Retrospective design introduced selection bias and constrained causal inference. Small sample size (n = 106) may compromise statistical power and generalizability. Tissue microarrays with 2-mm cores restricted spatial analysis, possibly missing heterogeneity. |
| Interpretation | 20 | Give a cautious overall interpretation of results considering objectives, limitations, multiplicity of analyses, results from similar studies, and other relevant evidence | 8 | The findings suggest PD-L1 expression patterns reflect immune microenvironment heterogeneity in tumor budding areas, with potential clinical relevance for predicting immunotherapy response, but are limited by the study's shortcomings. |
| Generalisability | 21 | Discuss the generalisability (external validity) of the study results | 8 | Tumor budding has been shown to be a predictor of LNM in cervical cancer and other early-stage cancers. Tumors with MT PD-L1 expression showed more favorable clinicopathologic characteristics. Marginal expression has been associated with better prognosis in some cancers and was more common in human papillomavirus (HPV)-associated tumors  Future research should prioritize evaluation of PD-L1 expression patterns as predictive biomarkers for immunotherapy response in larger cohorts. |
| Other information | |  | | |
| Funding | 22 | Give the source of funding and the role of the funders for the present study and, if applicable, for the original study on which the present article is based | 9 | Supported by grants from the Social Development Science and Technology Project of Meizhou, China (2022B11), and the Longgang Medical Discipline Construction Fund, China. Funders had no role in study design, data collection and analysis, decision to publish, or manuscript preparation. |

*Give information separately for cases and controls in case-control studies and, if applicable, for exposed and unexposed groups in cohort and cross-sectional studies.

**Note:** An Explanation and Elaboration article discusses each checklist item and gives methodological background and published examples of transparent reporting. The STROBE checklist is best used in conjunction with this article (freely available on the Web sites of PLoS Medicine at http://www.plosmedicine.org/, Annals of Internal Medicine at http://www.annals.org/, and Epidemiology at http://www.epidem.com/). Information on the STROBE Initiative is available at www.strobe-statement.org.
